# Letter from L. R. Lincecum, M.D., of Long Point, Texas

**Published:** 1874-04

**Authors:** L. R. Lincecum

**Affiliations:** Long Point, Texas


### CORRESPONDENCE.

Long Point, Texas, March 1, 1874.

*To The Southern Medical Record:*

Mrs. J. G., aged 22 years, married, her first child is a year and two month old, and at 5 o'clock this morning she was delivered of a very singular, and badly deformed seven months fœtus.

When I arrived at 4 o'clock A. M., she said she had been in labor since midnight. The symptoms being urgent. I immediately proceeded to make an examination; found a face presentation. After rectifying that error in the process, the labor went on naturally, and without further difficulty, until the successful delivery of the fœtus and a shapeless mass or mole, having an irregular surface and weighing three and a half pounds. There was no undue quantity of fluid or unusual hemorrhage presented in the case. With the mother everything proceeded as favorably as could be desired. But the fœtus and its malformation, is a matter of a different character. A case of deformity, that, if I shall succeed in presenting a clear description, may interest the curious investigator. The head, face, arms and lower extremities, all natural and full size for the period of its growth; neck short; upper portion of the spinal column curved, giving the thorax a depressed and shortened figure; posteriorally and opposite the superior portion of the sacrum, was a wen-like, rather soft, shapeless mass, as large as an ordinary biscuit. In front, and arising from the umbilical region, slightly adhering to and spreading to the left, on the surface of the lower portion of the thorax lay one entire lobe of the lungs. Protruding from the same aperture, and not adhering to anything outside, were the entire amount of the small intestines. Then came the liver, spread upon and partly adhering to the external surface of the abdomen, and extending downwards to the pubic region.

It was a female fœtus, and from the greatly deformed, tumefied, condition of the genital fisure, seeming to be an elongation of the clitoris, came the umbilical cord. This appeared healthy and of the ordinary length. The entire pubic region and external genital organs were much enlarged and distorted—a perfect monstrosity.

At the moment of the birth the fœtus manifested indications of life, but it made no effort to breathe—the breathing aparatus seemed to be altogether out of position—the vital spark ceased to burn with the delivery of the placenta, which immediately followed the birth of the child.

The mother had been healthy during the period of gestation; but she is an extremely sympathetic affectionate wife; her husband struggling in the last stage of pulmonary consumption. To my interogatory, "have you been hurt or frightened at any time?" Her reply was no, but every nerve in my system has been so constantly rasped and irritated, by the deep, hollow, rattling cough of my declining, dear husband, that, hurts or frights could not have done more.

March 3d. Patient cheerful and doing well every way, but has too much milk—procured a young lamb to draw her breasts. It performs very satisfactory.

March 5th. Patient says it looks like laziness to be lying abed, feeling as well as she does.

March 12th. Patient up and says she is in better health than is usual with her.

You are at liberty to dispose of this acticle as you may think proper. L. R. Lincecum, M.D.

